# Chirurgie des hyperthyroïdies: à propos de 60 cas

**DOI:** 10.11604/pamj.2018.31.43.16695

**Published:** 2018-09-20

**Authors:** Youssef Darouassi, Mohamed Amine Hanine, Abdelfettah Aljalil, Amine Ennouali, Brahim Bouaity, Mohamed Mliha Touati, Haddou Ammar

**Affiliations:** 1 Service d'Oto-Rhino-Laryngologie, Hôpital Militaire Avicenne, Marrakech, Maroc

**Keywords:** Hyperthyroïdie, chirurgie, thyroïdectomie, Hyperthyroidism, surgery, thyroidectomy

## Abstract

L'hyperthyroïdie englobe plusieurs maladies caractérisées par un niveau élevé d'hormones thyroïdiennes circulantes. La thyroïdectomie en est un des principaux traitements. L'objectif de notre étude est d'évaluer, à travers une étude rétrospective, les particularités épidémiologiques, cliniques, thérapeutiques, et évolutives des patients ayant bénéficié d'une prise en charge chirurgicale d'une hyperthyroïdie dans le service d'ORL à l'hôpital militaire Avicenne de Marrakech. Soixante patients ont été colligés avec une prédominance féminine et une moyenne d'âge de 52 ans. La clinique a été dominée par les signes de thyrotoxicoses qui ont été retrouvés chez tous les patients. L'exploration a permis d'identifier 47 cas de goitre multihétéronodulaire toxique ou prétoxique (soit 78.33% des cas), 5 cas de maladies de Basedow (soit 8.33%) et 8 cas d'adénome toxique (soit 13.33%). Une préparation médicale a été de mise chez tous nos patients. Une thyroïdectomie totale a été réalisée chez 50 patients (soit 83.33%) et une loboisthmectomie chez 10 patients (soit 16.33%). En post-opératoire, des complications sont été colligés: un cas de paralysie laryngée transitoire (1,6% des cas), neuf cas d'hypoparathyroïdie transitoire (15% des cas), un cas d'hypoparathyroïdie définitive (1,6% de cas), et un seul cas d'hématome compressif (1,6% de cas). La chirurgie des goitres toxiques réputés hémorragiques et adhérents, doit être réalisée par un chirurgien expérimenté qui doit doubler de vigilance pour minimiser la morbidité représentée essentiellement par la paralysie laryngée et l'hypoparathyroïdie.

## Abstract (en)

Hyperthyroidism includes several diseases characterized by high level of circulating thyroid hormones. Thyroidectomy is one of the main treatment options. We conducted a retrospective study to evaluate the epidemiological, clinical, therapeutic, evolutionary features of patients who had undergone surgery for hyperthyroidism in the ENT Department at the Avicenne Military Hospital in Marrakech. Data were collected from medical records of 60 patients. There was a female predominance and the average age was 52 years. Clinical manifestations were dominated by thyrotoxicosis in all patients. Clinical examination allowed the identification of 47 cases of toxic or pretoxic multiheteronodular goitre (78.33% of cases), 5 cases of Graves disease (8.33% of cases) and 8 cases of toxic adenoma (13.33% of cases). All patients underwent medical preparation. Total thyroidectomy was performed in 50 patients (83.33%) and loboisthmectomy in 10 patients (16.33%). Postoperative complications were: transient laryngeal paralysis in one case (1.6%), transient hypoparathyroidism in nine cases (15%), definitive hypoparathyroidism in one case (1.6%) and compressive hematoma in one case (1.6% of cases). Surgical treatment of bleeding and adherent toxic goiters should be performed by an experienced surgeon who must remain vigilant to minimize morbidity mainly caused by laryngeal paralysis and hypoparathyroidism.

## Introduction

L'hyperthyroïdie est un hyperfonctionnement de la glande thyroïde conduisant à un état de thyrotoxicose pouvant être responsable de possibles complications notamment cardiaques et ophtalmiques. Le traitement de l'hyperthyroïdie repose essentiellement sur les antithyroïdiens de synthèse, l'iode radioactif et la chirurgie. Le but de cette dernière est d'obtenir une euthyroïdie avec le minimum de morbidité récurrentielle et parathyroïdienne. Le traitement chirurgical est globalement dominé par la thyroïdectomie totale alors que l'isthmo-lobectomie reste l´apanage des nodules toxiques isolés. L'objectif de notre étude est d'évalué les caractéristiques épidémiologiques, cliniques, thérapeutiques, et évolutives des patients ayant bénéficié d'une prise en charge chirurgicale d'une hyperthyroïdie dans le service d'ORL de l'hôpital militaire Avicenne Marrakech.

## Méthodes

Il s'agit d'une étude rétrospective étalée sur 5 ans, allant de janvier 2009 à décembre 2013, portant sur 60 dossiers de patients opérés pour une hyperthyroïdie. Nous avons inclus dans cette étude les patients opérés dans le service pour nodule thyroïdien toxique, goitre thyroïdien multi-nodulaire toxique, maladie de Basedow ou goitre prétoxique. Nous avons exclu de cette étude les dossiers incomplets et inexploitables. Pour la réalisation de notre travail, nous avons élaboré une fiche d'exploitation comprenant les différentes données épidémiologiques, cliniques, paracliniques, thérapeutiques, anatomopathologiques et évolutives. En ce qui concerne les considérations éthiques, le recueil des données a été effectué avec respect de l'anonymat des patients et de la confidentialité de leurs informations.

## Résultats

**Figure 1:**
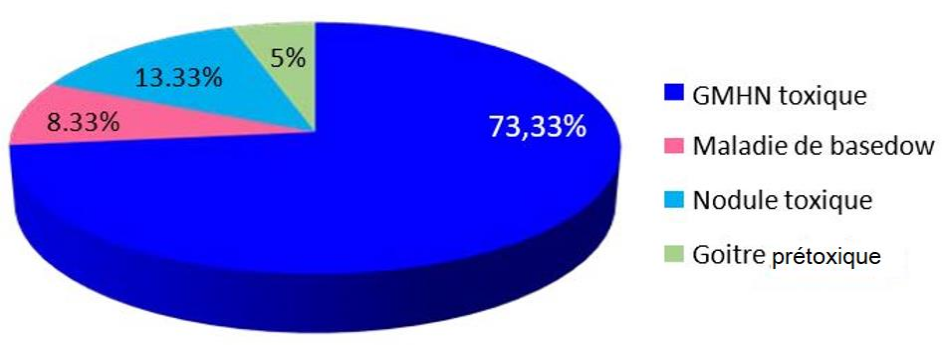
Les étiologies des hyperthyroïdies opérées

**Figure 2:**
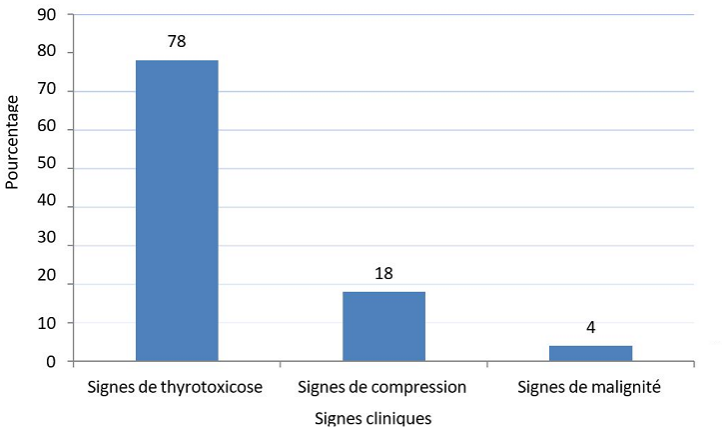
Répartition des signes cliniques

Sur 591 cas opérés entre janvier 2009 et décembre 2013, 60 cas se sont révélés en hyperthyroïdie (soit 10,15%) des cas. Sur 60 cas, nous avons trouvé 52 femmes (soit 86,66%) et 8 hommes (soit 13,33%), soit une sex-ratio (homme/femme) de 0,15. L'âge moyen de nos patients était de 46 ans avec des extrêmes allant de 18 à 69 ans. La tranche d'âge la plus touchée se situait entre 38 et 47 ans avec une fréquence de 44.44%. Neuf patients avaient des antécédents familiaux de nodule thyroïdien bénins opérés. Les étiologies des hyperthyroïdies ont été répartit en: 44 goitres multihétéronodulaire (soit 73,3%), cinq cas de maladie de basedow (soit 8,3%), huit cas de nodule toxique (soit 13,3%) et trois goitres thyroïdiens pré toxiques (soit 5%) (Figure 1). Les signes fonctionnels ont été dominés par un tableau clinique de thyrotoxicose. Les signes de compressions, et les signes évocateurs de malignités ont été retrouvés dans quelques cas (Figure 2). Les signes fonctionnels de thyrotoxicose retrouvés à l'examen clinique sont résumés dans le Tableau 1. Onze patients présentaient des signes de compressions: tous nos patients ont bénéficié d'une laryngoscopie indirecte qui a objectivé une parésie de la corde vocale droite chez l'un de nos deux patients dysphoniques. Une échographie cervicale a été systématiquement réalisée chez tous nos patients, en montrant un goitre homogène dans 2 cas (soit 3,33%), un nodule unique chez 5 patients (soit 8,33%), un goitre multinodulaire dans 53 cas (soit 88,33%). L'aspect échographique retrouvé: lésions hypoéchogènes chez 16 patients (soit 26.56%), lésions hétérogènes dans 17 cas (soit 28.12%), microcalcifications dans 4 cas (soit 6,66%) et Des nodules homogènes chez 6 patients (soit 10). Trois patients ont bénéficié d'un scanner cervico thoracique pour goitre plongeant.

**Tableau 1:** Répartition des signes cliniques de thyrotoxicose

Signes cliniques	Fréquence (%)
Tachycardie	78,3
Asthénie	68,3
Amaigrissement	39,5
Irritabilité	38,9
Hypersudation	20,7
Tremblement	21,3
Thermophobie	15,1
ACFA	8,33
Exophtalmie	6,8

**Figure 3:**
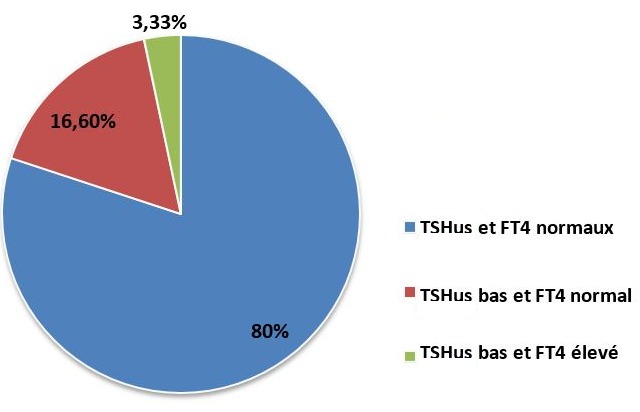
Répartition des taux de TSHUS et FT4 après préparation

Une imagerie par résonance magnétique a été réalisée en complément du scanner chez 2 patients (soit 3,33%) pour préciser l'extension inférieure du goitre et les rapports vasculaires. Dix-huit patients de notre série ont bénéficié de scintigraphie thyroidienne: cinq cas (soit 8,33%) présentaient des nodules toxiques et un cas de fixation homogène a été retrouvé. L'ECG a été systématiquement réalisé chez tous nos patients et n'a objectivé aucune anomalie. Une écho-cardiographie réalisée chez 5 patients n'a objectivée aucune anomalie. Tous les patients de notre série ont bénéficié d'une préparation médicale assurée par un endocrinologue en vue d'opérer un malade en euthyroïdie clinique et biologique. Cette préparation a été à base d'ATS associés ou non à un bêtabloquant ou à la L thyroxine. La durée moyenne de prise des antithyroïdiens de synthèses était de 6 mois avec des extrêmes allant d'un mois à plus de 40 mois. Après préparation médicale, en préopératoire: quarante-huit patients (soit 80%) avaient des taux de TSHus et de FT4 normaux, dix patients (soit 16,6%) avaient un taux de TSHus diminuée et un taux de FT4 normal et deux patients (soit 3,33%) avaient un taux de TSHus diminué et un taux de FT4 élevé (Figure 3). Une thyroïdectomie totale a été réalisée dans 50 cas (soit 83,33%): 40 cas de GMHN toxiques, 8 cas de maladie de basedow et 2 goitres pré toxiques. Une loboisthmectomie a été réalisée dans 10 cas de nodule toxique (soit 16,66%). L'étude histologique avait conclu à la bénignité dans 98,33% des cas. Un carcinome vésiculaire a été découvert dans un seul cas (soit 1,67%). Les suites postopératoires étaient simples chez 42 patients (soit 70%). Un traitement à base d'antalgiques seuls a été prescrit en postopératoire. L'ablation du drain a été réalisée en général à J2-J3. La durée moyenne d'hospitalisation était de 4 jours. Un seul cas (soit 1,6%) de paralysie récurrentielle unilatérale transitoire a été observé, bien compensé après une rééducation orthophonique. L'hypocalcémie aigue a été observée chez 10 patients (soit 16,6%), dont 6 cas présentaient une hypocalcémie fruste et 4 cas d'hypocalcémie patente qui a été jugulée par la supplémentation en calcium. Un seul cas (soit 1,6%) d'hypoparathyroïdie permanente a été observé. Aucun cas de paralysie récurrentielle bilatérale n'a été observé. Un hématome de la loge thyroïdienne a été noté dans un cas (soit 1,6%) nécessitant une reprise chirurgicale en urgence.Tous les patients ont été adressés au service d'endocrinologie de l'Hôpital Militaire Avicenne Marrakech pour opothérapie ainsi que le cas de carcinome vésiculaire qui a bénéficié d'un traitement adapté à base d'Iode radioactif.

## Discussion

Le sexe féminin est majoritaire dans l'ensemble des séries, avec un taux variant de 78,5% à 85,5% [1, 2]. La moyenne d'âge des patients opérés pour un goitre toxique est entre 40 et 50 ans [1, 3]. La moyenne d'âge de notre série était de 46 ans. Le syndrome de thyrotoxicose associe des signes généraux, cardiovasculaires, digestives, neuromusculaires, ophtalmologiques et psychiques [4]. Les signes de compressions à type de dyspnée, dysphagie ou de dysphonie sont à rechercher. Dix patients (soit 16,66%) de notre série ont présenté des signes de compressions. La laryngoscopie indirecte permet l'évaluation de la mobilité des cordes vocales avant la chirurgie. Dans notre série, cet examen a été réalisé systématiquement chez tous nos patients, et n'a pas objectivé d'anomalies. L'échographie est l'examen de référence pour l'analyse morphologique des nodules thyroïdiens et pour la détection de nodules infracliniques. Le compte-rendu doit décrire les caractéristiques des nodules (nombre, taille, échostructure, contours) et la présence d'adénopathies. Actuellement, Le système TIRADS occupe une place importante [5]. La scintigraphie thyroïdienne permet de différentier les nodules chauds des nodules froids. Elle permet également de préciser si un nodule est dominant dans un goitre multihétéronodulaire et de montrer une extension sous-sternale. La scintigraphie garde surtout sa place en cas de TSH basse notamment lorsque la thyroïde est multinodulaire pour identifier les nodules hyperfixants [6]. La radiographie thoracique peut montrer un élargissement du médiastin supérieur en rapport avec un goitre plongeant, et permet d'apprécier le retentissement sur la trachée. Le scanner cervicothoracique donne des renseignements précieux sur l'extension du goitre plongeant et le retentissement d'un volumineux goitre sur les éléments du cou dans le cadre du bilan d'extension ou de surveillance des cancers différenciés [7]. Sur le plan biologique, le dosage de la TSH est à réaliser en première intention. Dans les formes typiques d'hyperthyroïdie, la concentration de la TSH est effondrée, inférieure à 0,02mU/l. Le dosage de T4-libre et de T3-libre est à demander en cas d'anomalie de TSHus [8]. Un taux élevé de T4 ou de T3 associé à un taux faible ou nul de TSH définit l'hyperthyroïdie. La cytoponction thyroïdienne à l'aiguille fine a démontré sa contribution majeure pour sélectionner les nodules nécessitant une chirurgie et réduisant de ce fait les indications chirurgicales. Les résultats sont actuellement exprimés selon la classification Bethesda [9].

La prise en charge des états thyrotoxiques dépond du mécanisme de l'hyperthyroïdie [10]. Le traitement médical repose d'abord sur les antithyrodïens de synthèse (ATS) dont seul le carbimazole est disponible au Maroc. Le traitement est prolongé pendant au moins 18 mois avec une phase d'attaque de 4 à 6 semaines jusqu'à la réduction de l'hyperthyroïdie; suivie d'une phase d'entretien soit en diminuant progressivement la dose des ATS, soit en la maintenant et en ajoutant la levothyroxine à posologie substitutive limitant le passage en hypothyroïdie qui peut aggraver une ophtalmopathie préexistante [11]. D'autres méthodes peuvent être utilisées: l'iodure, le carbonate de lithium et le perchlorate de potassium ainsi que glucocorticoïdes, colestyramine, acide iopanoïque et ipodate, épurations extrarénale et plasmaphérèses [10]. La durée de prise des ATS avant la chirurgie dans notre série était en moyenne de 6 mois car il est recommandé autant que possible de n'intervenir que sur un patient en euthyroïdie. On prescrit dans les semaines qui précédent l'intervention des bêtabloquants qui diminuent les manifestations sympathicomimétiques de l'hyperthyroïdie, protègent des risques de crises thyrotoxiques préopératoires et diminuent le caractère vasculaire du goitre [12, 13]. Un délai de préparation beaucoup plus court est nécessaire en cas de situation d'urgence extra thyroïdienne (cardiaque ou ophtalmologique principalement), de goitre compressif, d'échec du traitement classique, ou d'intolérance aux antithyroïdiens de synthèse. Les bêtabloquants seuls ou associés à l'iodure de potassium permettant alors de réduire ce délai à une dizaine de jours, à condition d'une surveillance péri-opératoire rigoureuse du fait d'une grande variabilité de réponse d'un sujet à l'autre. Une autre préparation médicale rapide de 6 jours associant les corticoïdes à fortes doses aux ATS et à l'iodure de potassium est intéressante car n'utilise pas les bêtabloquants [14]. Dans notre série deux patients ont bénéficiés de protocoles de préparation rapide. L'usage de la radiothérapie métabolique dans l'hyperthyroïdie remonte aux années 1940-1950 avec comme principales contre-indications: la grossesse et l'ophtalmopathie basedowienne évolutive; une hypothyroïdie à court ou moyen terme peut survenir, nécessitant une supplémentation par levothyroxine et une deuxième dose est nécessaire si l'hyperthyroïdie persiste [10]. Aux Etats-Unis, l'iode radioactif (I131) constitue le traitement de première intention chez les patients atteints de la maladie de Basedow, alors qu'il est utilisé chez 25% de ces patients en Europe [15]. En cas de nodule toxique, l'I131 obtient la disparition de l'hyperthyroïdie dans 85% à 100% des cas [12]. Il est préférentiellement choisi chez le sujet âgé atteint de troubles de rythmes ou d'insuffisance cardiaque, ou sous anticoagulants. Mais il donne d'excellents résultats chez les sujets jeunes [10].

La chirurgie permet un traitement curatif radical. C'est le traitement de choix en cas de nodule toxique isolé et de GMHN toxique. En revanche, pour la maladie de Basedow, les indications sont: échec ou récidive après traitement médical, allergie aux ATS, goitre volumineux, nodules associés, comorbidités associées (diabète, cardiothyréose), désir de grossesse, mauvaise observance thérapeutique ou ophtalmopathie grave [10]. Mais la chirurgie est parfois difficile devant un goitre très vascularisé ou un aspect de thyroïdite fibreuse rendant difficile la dissection du récurrent [12]. Il est possible de limiter les saignement en ne liant les veines thyroïdiennes inférieures ou isthmiques qu'après ligature des artères thyroïdiennes pour maintenir le drainage sanguin [12]. En post opératoire, le traitement substitutif est indiqué en cas de thyroïdectomie totale. La totalisation isotopique a été effectuée après arrêt de L-thyroxine de 4 semaines ou en post chirurgical pour 15 de nos patients, et le nombre de cures était variable: 12 patients ont reçu une seule cure, 2 d'entre eux en ont reçu 2 et le dernier 3 cures. Sur le plan histologique, la proportion de goitres toxiques cancéreux est faible mais pas rare [16]. La surveillance est aussi bien commune à toute chirurgie thyroïdienne que spécifiques à la chirurgie de l'hyperthyroïdie. Il faut surveiller pouls, tension artérielle, température, respiration, phonation, crise thyréoprive et surtout le drain et la région cervicale antérieure. Au long cours, il faut rechercher des signes de complications latentes: hypothyroïdie, hypocalcémie.

## Conclusion

L'hyperthyroïdie n'est pas rare mais peut être grave par son retentissement. Sa prise en charge est multidisciplinaire. Trois situations peuvent être distinguées: l'adénome toxique, le goitre multihétéronodulaire toxique et la maladie de basedow. Les signes de suspicion cliniques permettent une orientation étiologique. De multiples examens paracliniques aident à orienter l'indication chirurgicale. L'échographie est l'examen de référence pour l'analyse des nodules thyroïdiens. La chirurgie trouve sa place surtout dans l'adénome toxique et le GMNH toxique. Alors que les indications sont limitées à certaines situations particulières dans la maladie de Basedow. La préparation médicale préopératoire et l'amélioration des moyens d'anesthésie et de réanimation ont nettement amélioré le pronostic de la chirurgie des goitres toxiques réputés hémorragiques et adhérents incitant le chirurgien à doubler de vigilance pour minimiser la morbidité surtout récurrentielle et parathyroïdienne.

### Etat des connaissances actuelles sur le sujet

- La chirurgie des hyperthyroïdies est réputée difficile;
- Une préparation médicale est nécessaire avant la chirurgie;
- Les indications sont actuellement bien codifiées.

### Contribution de notre étude à la connaissance

- Notre étude confirme que l'hyperthyroïdie n'est pas synonyme de bénignité;
- L'ECG réalisé systématiquement n'a montré aucune anomalie dans notre série.
